# Impact of the COVID-19 pandemic on brain death detection in German hospitals: a state-wide analysis of health data

**DOI:** 10.1186/s42466-025-00368-1

**Published:** 2025-02-24

**Authors:** Daniela Schoene, Martin Roessler, Katharina Eder, Albrecht Günther, Konrad Pleul, Axel Rahmel, Kristian Barlinn

**Affiliations:** 1 Department of Neurology, Faculty of Medicine and University Hospital Carl Gustav Carus, Technische Universität Dresden, Fetscherstraße 74, 01307 Dresden, Germany; 2 BARMER Institute for Health Care System Research (Bifg), Berlin, Germany; 3 Department of Neurology, University Hospital Jena, Jena, Germany; 4 Deutsche Stiftung Organtransplantation (DSO), Frankfurt Am Main, Germany

**Keywords:** COVID-19, COVID-19 pandemic, Brain death, Brain death detection, German hospitals

## Abstract

**Background:**

The low rate of organ donation in Germany has been linked to a deficit in the detection of patients with brain death (BD) in hospitals. It is unclear how crisis-related health system disruptions, such as the COVID-19 pandemic, affect this detection deficit.

**Methods:**

Secondary data analysis of anonymized data from deceased patients with acute brain injury from Saxony, Saxony-Anhalt and Thuringia during the pre-pandemic and pandemic period (01/2019–12/2022). Pandemic phases were stratified according to the predominant SARS-CoV-2 variant. Logistic multilevel models were employed to assess outcomes including diagnosis of BD, deceased organ donations, missed cases with potential BD and organ donation-related interactions with the German Organ procurement organization. Models accounted for regional COVID-19 incidence and first-dose vaccination rates, as well as age, gender and types of brain injuries.

**Results:**

A total of 11,100 deceased individuals from 136 hospitals were analyzed. An inverse association was observed between COVID-19 incidence and the determination of BD (adjusted odds ratio [aOR] 0.94, 95%CI [0.91; 0.97]; *p* < 0.001) as well as deceased organ donation (aOR 0.94, 95%CI [0.90; 0.98]; *p* = 0.001). When stratified by pandemic phases, this inverse association was evident for both BD determination (aOR 0.92, 95%CI [0.87; 0.99]; *p* = 0.02) and deceased organ donation (aOR 0.90, 95%CI [0.83; 0.97]; *p* = 0.01) during the initial wild-type phase. In the alpha phase, the association was observed only for BD determination (aOR 0.76, 95%CI [0.59; 0.98]; *p* = 0.03). No association was found in subsequent pandemic phases.

**Conclusion:**

The initial impact on BD detection during the pandemic highlights the importance of the health system's adaptive capacity in times of crisis.

**Supplementary Information:**

The online version contains supplementary material available at 10.1186/s42466-025-00368-1.

## Introduction

The COVID-19 pandemic significantly impacted the regulatory instruments of the healthcare system in Germany. To ensure continued care for patients with severe COVID-19, many hospitals converted inpatient units and expanded their intensive care capacities. However, studies have indicated a decline in the quality of care for patients with non-COVID-19 conditions, including cancer, stroke and cardiovascular disease [1–3]. Additionally, cohort studies from the United Kingdom and the United States have shown an increase in in-hospital mortality rates across all patient populations depending on intensive care bed occupancy [4, 5].

In Germany, organ donation and allocation are governed by the German Transplantation Act [6]. A fundamental prerequisite for organ donation is the determination of brain death (BD) prior to donation, as opposed to donation after circulatory death, which is not permitted under German law. Germany also follows an opt-in consent model, requiring individuals to explicitly consent to organ donation during their lifetime or through their next of kin, in contrast to the opt-out model, where consent is presumed unless explicitly denied. While not yet scientifically proven, these legal frameworks are believed to contribute to low donation rates in Germany. Countries implementing both donation after circulatory death and an opt-out model report donation rates nearing 50 per 1 million inhabitants, whereas Germany consistently reports lower rates of around 10 per 1 million (11.4 in 2023) [7, 8]. In addition, in-hospital organizational challenges may further impact donation rates. Recent analyses of German healthcare data indicate a lack of consistent identification of patients who might progress toward brain death during hospitalization, potentially overlooking individuals who could qualify as organ donors [9].

The organ donation situation has further worsened during the COVID-19 pandemic, as evidenced by an estimated 16% decrease in global transplant activity in the first year [10]. In Germany, the number of organ donors decreased by 29% and the number of deceased organ donations decreased by 28% in 2022 compared to the preceding year [11]. These changes may be attributed to uncertainties in handling SARS-CoV-2 infected donors [12]. However, existing deficiencies in identifying patients who may progress towards BD during hospitalization, might have worsened during the pandemic [9, 13]. Organ donation processes may have been deprioritized due to the focus on treating COVID-19 patients, changes in staffing responsibilities, increased workload, and restricted access to neurointensive care consultation services. Currently, no data is available on the identification of potential organ donors, a crucial step preceding deceased organ donation, during the COVID-19 pandemic.

Based on a secondary data analysis from three German states, we investigated whether the COVID-19 pandemic exacerbated the shortfall in identifying patients with irreversible cessation of brain function, who are potential organ donors, in procurement hospitals. The COVID-19 pandemic serves as a model for severe crisis affecting the healthcare system, representing future crisis scenarios, to examine the impact of external factors on organizational processes preceding organ donation.

## Methods

### Data source and study population

The data source comprised anonymized data from two primary sources: procurement hospitals in the federal states of Saxony, Saxony-Anhalt and Thuringia, and the German organ procurement organization (OPO; which in Germany is the Deutsche Stiftung Organtransplantation, DSO). These data were routinely merged to form the TransplantCheck dataset, as mandated under §11 of the German Transplantation Act [6]. Hospital-provided data were routinely collected as part of standard administrative and clinical documentation processes. Specifically, these data included all deaths where a primary and/or secondary brain injury was coded as a main or secondary diagnosis according to ICD-10-G. Additional variables included patient identifiers, demographic details (e.g., age and gender), whether mechanical ventilation was performed and its duration, type of brain injury and all coded primary and secondary diagnoses according to ICD-10-GM. The DSO provided complementary patient-level data on organ donation-related activities, including any documented interactions between procurement hospitals and the DSO regarding potential donors, whether BD was determined and the occurrence of deceased organ donation [14].

As mandated by the German Transplantation Act, TransplantCheck is routinely used to perform individual case analysis, identifying reasons why hospitalized patients, retrospectively deemed at risk of developing BD, were not subjected to a corresponding neurological evaluation, potentially missing cases [15]. Potentially missed BD cases were defined as those likely to progress to BD based on a retrospective review of all available clinical findings and brain imaging results, but where no BD evaluation was conducted, in the absence of documented reasons precluding further evaluation or organ donation (e.g., lack of imaging evidence of increased intracranial pressure, documented refusal of intensive care continuation or organ donation, denial by a proxy, or shift to palliative care due to medical reasons such as respiratory failure). This measure serves as an indicator of deficiencies in identifying potential donors in procurement hospitals [13]. TransplantCheck analyses are routinely conducted biannually jointly by transplant coordinators from the corresponding procurement hospital and the DSO.

For study purposes, only potential donors—defined as those who were mechanically ventilated at or near the time of death and had no coded diagnosis considered an absolute contraindication for organ donation (i.e., active malignancies except for specific brain tumors, uncontrolled infections, or transmissible diseases)—were included in the analysis [16, 17]. Prior to March 2022, cases with SARS-CoV-2 infections were excluded from analyses due to being considered a contraindication for organ donation. However, starting in March 2022, SARS-CoV-2 infection was no longer considered an absolute contraindication in Germany and was therefore included in analyses [18].

### Study endpoints

TransplantCheck dataset included the following procedural quality indicators of donor identification, which were used as study outcome measures:

1. Guideline-based determination of BD: the frequency of cases in which BD was diagnosed during hospitalization and reported to the DSO
2. Deceased organ donation: the frequency of cases in which organ donation was performed following BD determination
3. Potentially missed cases of BD: the frequency of patients, based on retrospective assessment of clinical findings and brain imaging, who were deemed likely to progress to BD but who did not undergo the necessary neurological evaluation
4. Organ donation-related interactions with the OPO: the frequency of documented contacts between procurement hospitals and OPO regarding potential donors, including notifications of suspected BD, requests for clinical support in BD determination and pre-assessment of donor eligibility

To capture continuous changes in study endpoints throughout the pre-pandemic and pandemic periods, the analysis considered only patients who died between January 1, 2019, and December 31, 2022. Phases of the pandemic were defined based on the predominant variant of SARS-CoV-2 and stratified according to data from the European Centre for Disease Prevention and Control (ECDC; i.e., Robert Koch-Institute) [19].

- January 1, 2019–January 26, 2020: Pre-pandemic
- January 27, 2020–February 1, 2021: Wild-type
- February 2, 2021–June 20, 2021: Alpha
- June 21, 2021–December 26, 2021: Delta
- December 27, 2021–February 28, 2022: Omicron I
- March 1, 2022–December 31, 2022: Omicron II (SARS-CoV-2 infection no longer an absolute contraindication)

### Regional COVID-19 incidence

Data on regional COVID-19 incidence were obtained from the Robert Koch-Institute and built upon the 2-week average of federal state-specific 7-day COVID-19 incidences per 100,000 inhabitants [20]. The use of the 2-week average accounted for expected delays between the reporting of COVID-19 cases and related hospitalizations.

### Confounder

The variables used to adjust for were defined as follows: age (in years), gender, type of brain injury (i.e., intracranial hemorrhage, ischemic stroke, hypoxic-ischemic encephalopathy, traumatic brain injury, encephalitis and infratentorial brain tumours) and the daily regional first-dose COVID-19 vaccination rate reported by the Robert Koch-Institute [21]. A detailed description of the operationalization of diseases based on hospital main and secondary diagnoses and the data sources used is provided in the supplementary material.

### Statistical analysis

For continuous variables, the median along with the 1st and 3rd quartiles were calculated. Categorical variables were described using absolute and relative frequencies along with their 95% confidence intervals (95% CI). Logistic multilevel models were employed to analyze associations adjusted for potential confounders between study endpoints and regional COVID-19 incidence rates. These models included a random intercept at the hospital site level to account for intra-cluster correlation. For anonymization purposes, dummy variables were assigned to the hospitals with the hospital identifier retained by the OPO. Study endpoints were treated as dependent variables, with the regional COVID-19 incidence rate as the independent variable. The logarithm base 2 of COVID-19 incidence rate was included in the models, allowing the adjusted odds ratio (aOR) to interpret the change in odds for each outcome with a doubling of COVID-19 incidence. *p*-values were provided as descriptive statistics. Potential confounders as stated above were accounted for in the models. The model for the expected value of outcome $Y_{ihr}$ of individual i treated in hospital h located in region r was: $E[Y_{ihr} |I_{r} ,V_{r} ,X_{ihr} ,\alpha_{h} ] = F^{ - 1} \left( {\mu + \gamma \cdot log_{2} \left( {I_{r} + 1} \right) + \eta \cdot V_{r} + X_{ihr}^{\prime } \beta + \alpha_{h} } \right)$,

where F() is the logistic link function, $\mu$ is a constant, $I_{r}$ is the regional 2-week average COVID-19 incidence with parameter $\gamma$, $V_{r}$ is the regional first-dose COVID-19 vaccination rate with parameter $\eta$, $x_{ihr}$ is the vector of individual-specific confounders with coefficient vector $\beta$, and $\alpha_{h}$ is a normally distributed random effect at the hospital level with mean 0 and variance $\sigma^{2}$. Note that pre-pandemic COVID-19 incidence rates were transformed before logarithmization by adding 1 to avoid taking the log of 0. To account for potential changes during the pandemic, separate multilevel models were estimated for the defined pandemic phases. To illustrate the strength of associations, probabilities were predicted for a representative female patient with average age and prevalence of brain injury types at the 10% (Q10) and 90% (Q90) quantiles of COVID-19 incidence during specific pandemic phases of interest. Statistical analyses were performed using R (Version 4.3.2).

### Ethics

The study was approved by the ethics committee of the Technical University of Dresden (BO-EK-222052022).

## Results

**Fig. 1:**
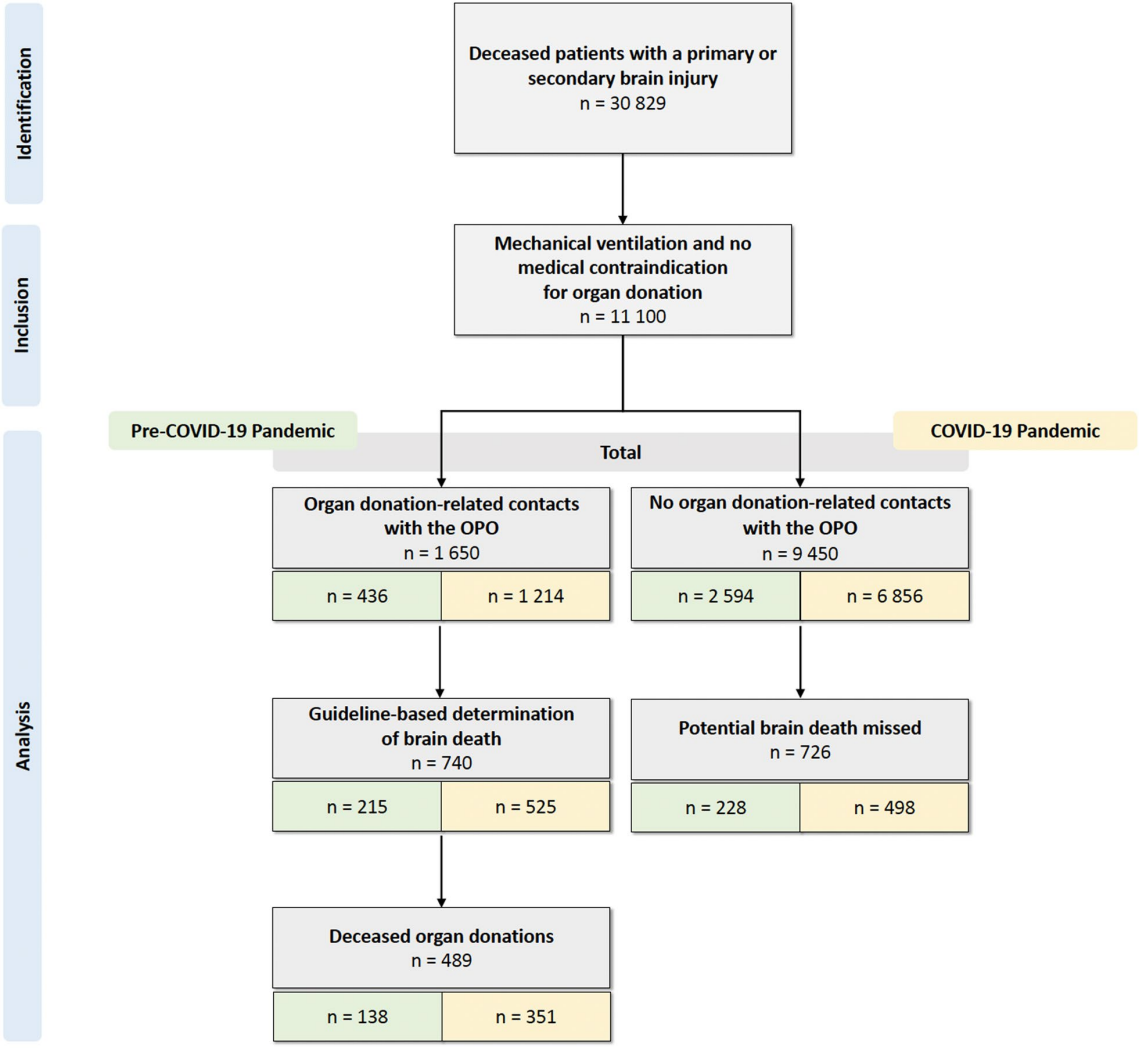
Flow chart of the study population

A total of 30,829 patients with a diagnosis of primary and/or secondary brain injury died in hospitals in the three federal states of Saxony, Thuringia and Saxony-Anhalt during the study period. Of these, 11,100 patients from 136 hospitals met the inclusion criteria and were included in the final analysis (Fig. 1). The most frequent types of brain injury were ischemic stroke (33.5%), intracranial haemorrhage (29.4%), hypoxic-ischemic encephalopathy (26.8%) and traumatic brain injury (17.6%). Further characteristics of the study population are listed in Table 1.

**Table 1:** Characteristics of the study population

Variable	Study population (n = 11,100)
Age, median (Q1;Q3)	70 (63;81)
Gender, n (%)	
Female	4487 (40.4)
Male	6613 (59.6)
Types of brain damage according to main or secondary diagnosis, n (%)	
Ischemic stroke	3723 (33.5)
Intracranial hemorrhage	3261 (29.4)
Hypoxic-ischemic encephalopathy	2970 (26.8)
Traumatic brain injury	1953 (17.6)
Other	300 (2.7)

n: Number of observations; Q1: lower quartile (25th percentile); Q3: upper quartile (75th percentile)

During the pandemic, BD was confirmed in 525 out of 8070 cases (6.5%; 95% CI [6.0; 7.1]), compared with 215 out of 3030 cases (7.1%; 95% CI [6.2; 8.1]) in the preceding period. The rate of deceased organ donation during the pandemic was 4.4% (351/8070; 95% CI [3.9; 4.8]) compared with 4.6% (138/3030; 95% CI [3.9; 5.4]) before the pandemic. In 498 out of 8070 cases (6.2%; 95% CI [5.7; 6.7]), individual case evaluations suggested that considering the severity of brain injury and the constellation of clinical findings, BD may have occurred during the course of the disease, without identifiable reasons for the lack of neurological evaluation. In the preceding period, this rate was 228 out of 3030 cases (7.5%; 95% CI [6.6; 8.5]). Organ donation-related interactions with the OPO were recorded for 1214 cases (15%; 95% CI [14.3; 15.8]) during the pandemic, compared to 436 cases (14.4%; 95% CI [13.2; 16.1]) before the pandemic. The organ donation realization rate (deceased organ donors / cases with confirmed BD) was 66.9% (351/525; 95% CI [62.7; 70.8]) during the pandemic, compared to 64.2% (138/215; 95% CI [57.6; 70.3]) before the pandemic.

**Fig. 2:**
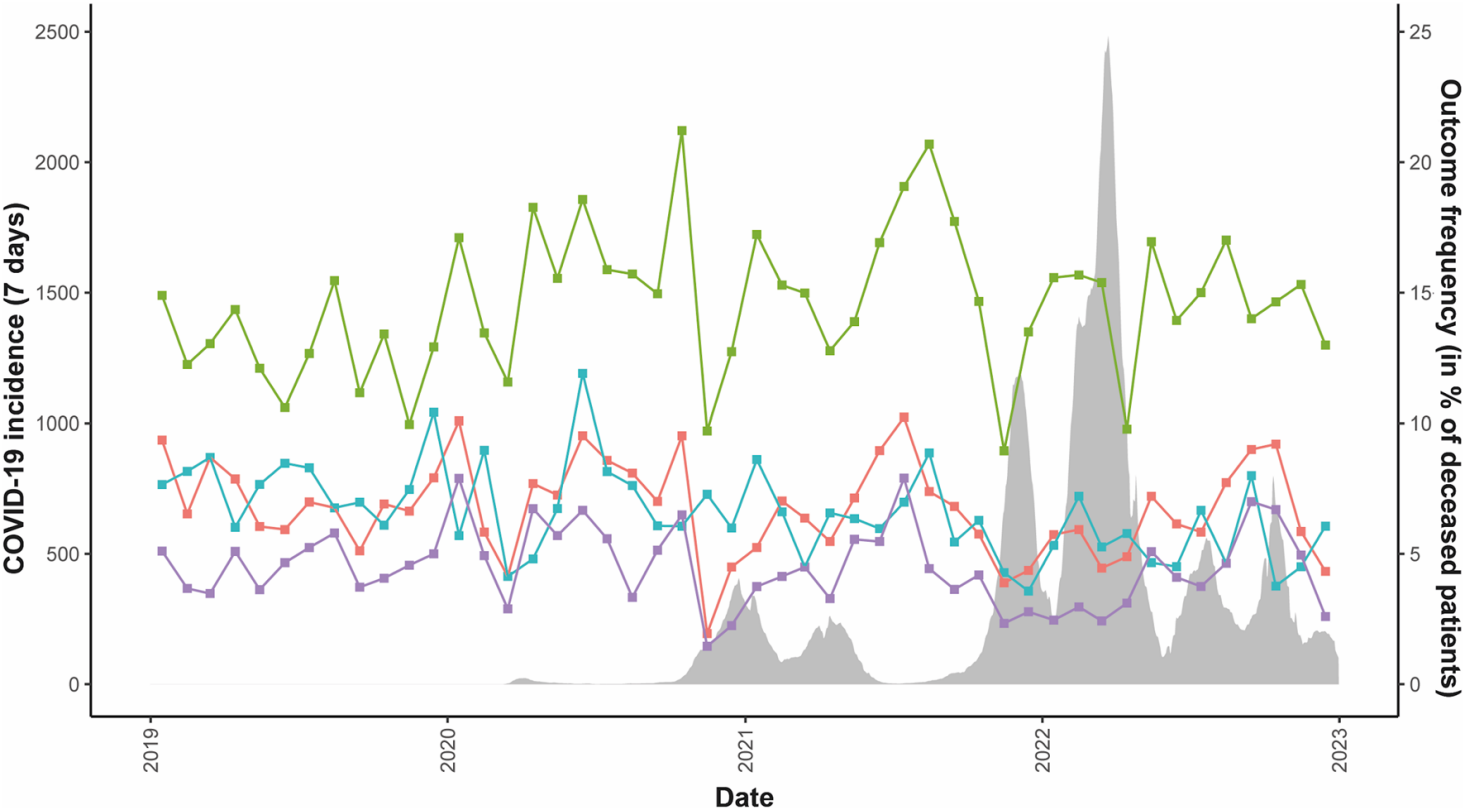
Monthly values for study endpoints in the context of population-weighted average COVID-19 7-day incidence (gray) in the federal states of Saxony, Thuringia and Saxony-Anhalt. *Orange graph*: diagnosis of brain death (BD), *purple graph*: deceased organ donation, *turquoise graph*: missed cases with potential BD, *green graph*: organ donation-related interactions with the German organ procurement organization

Figure 2 illustrates the study endpoints alongside regional COVID-19 incidence over time. A notable decline in cases with diagnosis of BD and in cases where potential BD was unrecognized is particularly evident at the onset of the second wave in autumn 2020 and the fourth wave in autumn 2021. Between these waves, a temporary increase in the frequencies of both study endpoints can be observed.

**Fig. 3:**
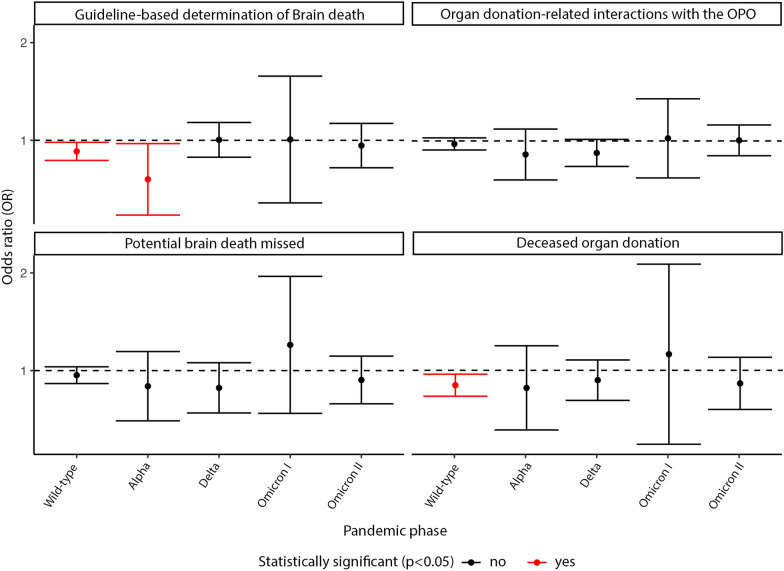
Overview of all adjusted odds ratios with their 95% confidence intervals for respective study endpoints relative to doubling of COVID-19 incidence rates during each pandemic phase (based on the predominant variant of SARS-CoV-2). Adjustments were made for age, gender, selected types of brain injuries and the federal state-specific COVID-19 first-dose vaccination rates

Considering the overall study period, regression analysis revealed a significant inverse association between regional COVID-19 incidence and the identification of BD (aOR 0.94, 95%CI [0.91; 0.97]; *p* < 0.001) as well as the number of deceased organ donation (aOR 0.94, 95%CI [0.90; 0.98]; *p* = 0.001). Upon stratification by pandemic phases, during the initial phase (wild-type), a significant inverse association was found between regional COVID-19 incidence and both BD identification (aOR 0.92, 95%CI [0.87; 0.99]; *p* = 0.02) and deceased organ donation (aOR 0.90, 95%CI [0.83; 0.97]; *p* = 0.01). In the second phase (alpha), an inverse association was observed only for BD identification (aOR 0.76, 95%CI [0.59; 0.98]; *p* = 0.03). No statistically significant associations were found for the endpoints of potentially missed cases of BD and organ donation-related interactions with the DSO. Detailed estimates of these associations for all study endpoints and pandemic phases are presented in Fig. 3.

During the wild-type phase, predicted probabilities for BD identification and deceased organ donation decreased with increasing regional COVID-19 incidences, ranging from 6.6% at Q10 (0 cases per 100,000 inhabitants) to 3.6% at Q90 (243 cases per 100,000 inhabitants) and from 5.1% at Q10 to 2.3% at Q90, respectively.

## Discussion

This large-scale secondary data analysis highlights the impact of the COVID-19 pandemic on procedural quality indicators related to the identification of potential organ donors in German procurement hospitals. Specifically, decreases in the rates of diagnoses of BD and deceased organ donations were notable during the wild-type and alpha phases of the pandemic. Interestingly, no correlation between these indicators and COVID-19 incidence was observed in subsequent pandemic phases, suggesting potential adaptive structural adjustments within hospitals. While the decreases in BD identification and organ donations were statistically significant, the overall impact of COVID-19 was modest, as indicated by an adjusted odds ratio of 0.94. However, even small changes in BD identification are relevant, as it is a rare event, and any shift in its identification may have an impact on organ donation processes.

International studies indicate a decline in the number of deceased organ donations during the COVID-19 pandemic [22–24]. However, data on the in-hospital identification of at-risk cases for BD, including both recognized and missed cases, are lacking. This is particularly relevant in countries where the confirmation of BD is a prerequisite for deceased organ donation, as unrecognized cases could have significantly impacted the organ donation landscape [9, 13]. A comprehensive analysis of secondary data from 128 German hospitals in 2016 showed that 6.3% of all deceased cases with acute brain injuries were expected to progress toward BD [13], yet neurological evaluation for BD determination was not performed in these cases for various reasons. Another study found that although the number of potential organ donors in Germany increased by 13.9% between 2010 and 2015, the actual number of organ donations decreased by 32.3% during the same period [9]. This discrepancy suggests that inadequate identification of potential donors in hospitals may be a significant factor contributing to the persistently low organ donation rates in Germany. The COVID-19 pandemic may have exacerbated this deficiency due to infection prevention measures, particularly since organ donation was initially contraindicated in patients with SARS-CoV-2 infection [18].

Labor-intensive measures and psychological distress likely compromised care quality during the pandemic, contributing to suboptimal treatment of isolated SARS-CoV-2 patients. A survey from Spain found that nearly 50% of healthcare professionals perceived a decline in care quality during the first wave, mainly due to increased workload and patient complexity [25]. Sleep disturbances, common during the crisis, may have further impaired frontline workers’ ability to deliver quality care [26]. Physicians, facing heightened stress from increased patient loads and fear of contracting COVID-19, reported significantly higher anxiety levels, particularly those treating more COVID-19 patients [27]. In a survey study of 135 physicians, over 70% expressed concern about their families contracting the virus, increasing stress and potentially distracting from patient care [28]. These factors likely influenced patient care, leading to the possibility of suboptimal identification of patients at-risk for BD. Medical challenges, such as performing the apnea test on SARS-CoV-2-infected or critically ill COVID-19 patients on ECMO therapy, may have posed additional barriers [29, 30]. However, it appears that awareness for BD identification was not generally restricted during the pandemic. Despite fluctuations, particularly during the alpha and wild-type phases, organ donation-related interactions with the OPO remained above 10% that aligns with the national average of 2010 to 2015, when the contact rate decreased from 11.5 to 8.2% [9]. Once BD was confirmed, subsequent organizational processes did not seem to be impaired; the donation realization rate during the pandemic did not appear compromised compared to the pre-pandemic period. Thus, the primary obstacle seems to lie in identifying patients who might potentially progress toward BD. It is also plausible that fewer patients were admitted to hospitals during the early phase of the pandemic and possibly at the onset of subsequent infection waves. This included patients with brain injuries who could potentially progress to BD. A nationwide cohort study in Germany involving 1463 hospitals reported a 17.4% decline in patients with acute ischemic stroke and a 15.8% decline in patients with intracerebral hemorrhage in the early months of the pandemic compared to pre-pandemic months [31]. Underlying reasons could include reduced perception of symptoms due to social restrictions and limited access to healthcare facilities because of lockdown measures. Furthermore, hospital admissions were more tightly regulated and pre-hospital triage criteria were made more stringent, as evidenced by a retrospective cohort study during the early months of the pandemic, which reported an almost 50% decrease in neurosurgical emergencies related to cranial or brain injuries compared to pre-pandemic levels [32].

During the pandemic, potential cases of BD may have been overlooked in 6.2% of all deceased patients. Detailed analysis across various phases consistently showed monthly non-recognition rates exceeding 5%. These findings are consistent with pre-pandemic data where potential BD was not identified in 6.3% of cases [13]. The persistently high rate of missed potential BD cases highlights ongoing challenges in early intra-hospital recognition and exacerbated deficiencies within procurement hospitals during the pandemic in Germany. The specific reasons for these missed cases during the pandemic have not been systematically investigated. Intra-hospital triage processes and additional infection control measures likely prolonged evaluation times, delaying prompt BD determination and leading to cases that might have been identified under normal circumstances being overlooked. For example, an observational study observed a significant increase in the time from patient admission to BD determination during the pandemic compared to pre-pandemic [22].

To enhance the resilience of healthcare systems during unforeseen global crises, such as the COVID-19 pandemic, several strategies can be implemented. Ezzati et al. emphasize the importance of learning from both national and international challenges to improve organizational resilience [33]. Key factors for enhancing resilience include promoting continuous improvement, strengthening leadership and ensuring effective resource management [33]. Integrating mental health services into primary care may help mitigate psychological stress, thus improving overall system resilience [34]. Beyond healthcare worker well-being, interventions such as training, telemedicine and optimizing workplace organization may contribute to enhancing hospital resilience [35]. In the context of BD identification, automated digital screening tools may aid in identifying potential organ donors among intensive care patients [36].

To the best of our knowledge, the present study represents the first state-wide analysis of the impact of the COVID-19 pandemic on organizational processes of organ donation in hospitals across Germany. The study focused on the states of Saxony, Saxony-Anhalt and Thuringia, which serve as a representative sample. In 2022, these states exhibited a deceased organ donation rate of 13.4 per 1 million population, surpassing the national average of 10.3 per 1 million [37]. The methodological approach using multilevel models allowed for a detailed examination, accounting for various pandemic phases and dynamics of SARS-CoV-2 spread.

Limitations of this analysis include potential biases from unmeasured confounders and the omission of actual ICU bed occupancy data. Nonetheless, hospital hygiene measures also affected areas beyond intensive care units, substantiating COVID-19 incidence as a proxy for overall hospital burden and enabling a thorough evaluation of organizational processes in identifying potential donors. Regional differences in healthcare provision may reduce the study's external validity. Data were included only until the end of 2022, as subsequent reductions in reporting SARS-CoV-2 incidences by the Robert Koch-Institute would have compromised data comparability. A further limitation is that individual case analyses were subjective and dependent on the experience and specialty of the transplant coordinators at each hospital. The assessment of whether a patient at risk of developing brain death was potentially overlooked relied on the decisions made by the involved transplant coordinators. Since these evaluations were retrospective, it is possible that reasons for early termination of therapies were not documented or that discussions with family members—where, for example, a patient’s refusal to donate organs may have been mentioned—were not recorded, leading to the absence of a brain death evaluation. This could have potentially resulted in an overestimation of the number of cases that were missed. Although such bias likely applies to the pre-pandemic period as well, variations inherent in these evaluations may explain why the absolute number of potentially missed cases were slightly higher in the pre-pandemic period. Conversely, brain death identification during the pandemic may have been underestimated, as SARS-CoV-2 was considered an absolute contraindication for organ donation until March 2022 and potential donors who died due to COVID-19 may have been missed.

## Conclusions

This study emphasized that the German healthcare system, while generally well-organized, reveals challenges in adapting quickly during crises. Targeted data analyses are crucial to enhance future crisis resilience. Ensuring that hospitals consistently identify potential organ donors is essential to provide critically ill patients continued opportunities for life-saving organ donations.

## Supplementary Information

Additional file1.

## Data Availability

The datasets used and analyzed during the current study are available from the corresponding author on reasonable request.

## Abbreviations

CI: Confidence interval
COVID-19: Coronavirus Disease 2019
ECDC: European Centre for Disease Prevention and Control
ECMO: Extracorporeal Membrane Oxygenation
OPO: Organ Procurement Organization
SARS-CoV-2: Severe Acute Respiratory Syndrome Coronavirus 2
