# Supplementary material for: Impact of the COVID-19 pandemic on brain death detection in German hospitals: a state-wide analysis of health data
Source: Neurol Res Pract. 2025 Feb 24;7(1):9. doi: 10.1186/s42466-025-00368-1 (PMC11921974; doi:10.1186/s42466-025-00368-1)
Supplement: Supplementary file 1 — Additional file1. [file 42466_2025_368_MOESM1_ESM.docx]

**Supplementary material**

**Operational Definitions of Primary and Secondary Diagnoses**

The frequencies of the following severe primary and/or secondary brain injuries were identified as either primary or secondary diagnoses according to the following ICD codes:

- **C70.*:** Malignant neoplasm of meninges
- **C71.*:** Malignant neoplasm of brain
- **D33.*:** Benign neoplasm of brain and other parts of central nervous system
- **B00.3 – B00.4:** Herpesviral meningitis/encephalitis
- **G00.*:** Bacterial meningitis, not elsewhere classified
- **G01.*:** Meningitis in bacterial diseases classified elsewhere
- **G02.*:** Meningitis in other infectious and parasitic diseases classified elsewhere
- **G03.*:** Meningitis due to other and unspecified causes
- **G04.*:** Encephalitis, myelitis, and encephalomyelitis
- **G05.*:** Encephalitis, myelitis, and encephalomyelitis in diseases classified elsewhere
- **G06.0:** Intracranial and intraspinal abscess and granuloma
- **G07.*:** Intracranial and intraspinal abscess and granuloma in diseases classified elsewhere
- **G08:** Intracranial and intraspinal phlebitis and thrombophlebitis
- **G91.*:** Hydrocephalus
- **G93.0:** Brain cyst
- **G93.1:** Anoxic brain damage, not elsewhere classified
- **G93.5:** Compression of brain
- **G93.6:** Cerebral edema
- **G93.80:** Persistent vegetative state
- **G94.0 – G94.2:** Hydrocephalus in infectious and parasitic diseases classified elsewhere/ in neoplastic diseases/ in other diseases classified elsewhere
- **I60.*:** Subarachnoid hemorrhage
- **I61.*:** Intracerebral hemorrhage
- **I62.*:** Other nontraumatic intracranial hemorrhage
- **I63.*:** Cerebral infarction
- **I64:** Stroke, not specified as hemorrhage or infarction
- **I67.*:** Other cerebrovascular diseases
- **O22.5:** Cerebral venous thrombosis in pregnancy
- **S06.*:** Intracranial injury
- **S07.*:** Crushing injury of head
- **S08.8:** Traumatic amputation of part of head
- **S09.7 – S09.9:** Multiple injuries of head, other specified injuries of head, unspecified injury of head
- **S18:** Traumatic amputation at neck level
